# Conservation of the RNA Transport Machineries and Their Coupling to Translation Control across Eukaryotes

**DOI:** 10.1155/2012/287852

**Published:** 2012-05-15

**Authors:** Paula Vazquez-Pianzola, Beat Suter

**Affiliations:** Institute of Cell Biology, University of Bern, Baltzerstrasse 4, 3012 Bern, Switzerland

## Abstract

Restriction of proteins to discrete subcellular regions is a common mechanism to establish cellular asymmetries and depends on a coordinated program of mRNA localization and translation control. Many processes from the budding of a yeast to the establishment of metazoan embryonic axes and the migration of human neurons, depend on this type of cell polarization. How factors controlling transport and translation assemble to regulate at the same time the movement and translation of transported mRNAs, and whether these mechanisms are conserved across kingdoms is not yet entirely understood. In this review we will focus on some of the best characterized examples of mRNA transport machineries, the “yeast locasome” as an example of RNA transport and translation control in unicellular eukaryotes, and on the *Drosophila* Bic-D/Egl/Dyn RNA localization machinery as an example of RNA transport in higher eukaryotes. This focus is motivated by the relatively advanced knowledge about the proteins that connect the localizing mRNAs to the transport motors and the many well studied proteins involved in translational control of specific transcripts that are moved by these machineries. We will also discuss whether the core of these RNA transport machineries and factors regulating mRNA localization and translation are conserved across eukaryotes.

## 1. Introduction

RNA transport coupled with translation control is a crucial mechanism to target protein expression to specific regions of a cell or an organism. During transport, mRNAs associate with proteins that control every step in the mRNA life cycle. Together, mRNAs and proteins form large ribonucleoprotein (RNP) complexes in which different factors control assembly, stability, translation, and transport of localized mRNAs. Microtubules, microfilaments, and their motors then transport these complexes to their final destination. To achieve local protein synthesis at the final target site, translation of transported mRNAs must be repressed during their journey and then activated only once the mRNAs reach their final destination.

Although several proteins involved in translation control of localized transcripts have been described, how translation repression during transport occurs and how local protein synthesis is activated at the final destination of a given mRNA are only partially known for a few mRNAs. In this paper we will focus on some of the best-characterized examples of translational regulation of localized transcripts and we will analyze whether the complexes regulating localization and translation are conserved in other eukaryotes. We will also attempt to shed light on the conservation of the coupling between mRNA localization and translational control across eukaryotes.

## 2. RNA Localization Supports Local Protein Synthesis from Bacteria to Metazoans

Mechanisms to target mRNAs to discrete subcellular locations, where their protein products are expressed locally, were traditionally thought to be a hallmark of eukaryotes, which synthesize and translate mRNAs in different cellular compartments, namely, the nucleus and the cytoplasm [1–4]. However, recent findings indicate that even in bacteria some mRNAs move from the nucleoid to particular regions of the cell before they get translated [5]. Most interestingly, a *Drosophila *transcript encoding a membrane protein was recently reported to localize to the membrane in *E. coli, *too [5]. This would imply that recognition of localizing signals within the transcripts have been conserved during evolution and with this probably large parts of the RNA transport machinery.

In unicellular eukaryotes such as yeast, many transcripts are actively transported to the cell bud. This is the case for *ASH1 *(asymmetric synthesis of HO) mRNA, which is localized to the bud of daughter cells and is essential for the mating-type switch (see what follows). RNA transport phenomenons have also been described in plants. In addition to cellular localization, transport of viral genomes, cellular mRNAs, and small RNAs (miRNAs and siRNAs) between cells through plasmodesmata and through the phloem is a common process in higher plants [6, 7]. Although the mechanisms regulating these processes are not well studied, it seems that cell to cell RNA movement is mediated by plant factors and that plasmodesmal transport is a highly regulated process. As an example, the homeodomain protein KNOTTED1 facilitates the transport of its own mRNA from cell-to-cell and this RNA is translated after its translocation [8, 9].

Some of the first examples of regulation of gene expression involving translational control of localized RNAs were described while studying embryonic development in metazoans. During this stage, maternal mRNAs accumulate in specific regions of *Xenopus* and *Drosophila *embryos, and translational control of these localized mRNAs is essential for embryogenesis. Examples of such process in *Drosophila *are the localization of the mRNA encoding the maternal determinant Bicoid (Bcd) to the anterior cortex of the oocyte, and of *oskar *(*osk*) and *nanos *(*nos*) mRNAs to the posterior cortex [10]. Their proper localization and translational control are essential for specifying the anteroposterior axis of the embryo. Similarly, localization of *gurken *(*grk*) mRNA to the dorsoanterior corner of the oocyte, next to the oocyte nucleus, is essential for the specification of the dorsoventral axis of the egg chamber and of the embryo [10]. In *Xenopus*, the mRNAs encoding the T-box transcription factor VegT and a member of the transforming growth factor-beta (TGF-*β*) family, Vg1, localize to the vegetal cortex of *Xenopus *oocytes and play roles in endodermal and mesodermal specification during early embryogenesis [11]. Importantly, a growing number of other mRNAs have also been reported to be localized in oocytes, eggs, and cleaving embryos of diverse organisms including the wasp *Nasonia vitripennis*, the cnidarian *Clytia hemisphaerica*, zebrafish, and several ascidian species, highlighting the importance of the RNA localization process across eukaryotes [12]. Other examples involve the accumulation and local synthesis of RNAs in the protruding edges of polarized cells, like fibroblasts and neurons [13, 14]. *β*-actin mRNA targeting to lamellipodia of chicken fibroblasts combined with its local translation at this site produces an enrichment of actin at the leading edge of these cells, a process that is required for cell motility [14]. *β*-actin mRNA is also localized in dendrites, where it is needed for ligand-dependent filopodial growth of rat hippocampal neurons [15, 16]. Some mRNAs are also localized and locally translated in axonal growth cones [13]. For example, local translation of *β*-actin mRNA facilitates Ca^2+^- and netrin-1- dependent growth cone guidance in *Xenopus *[17, 18].

Surprisingly, a systematic study of 3370 transcripts expressed during embryonic development in *Drosophila *showed that 71% of the analyzed mRNAs exhibited clear subcellular distribution patterns, suggesting that virtually all aspects of cellular function are impacted by RNA localization pathways [19]. Interestingly, many of these mRNAs showed novel patterns of localization, which suggest the existence of so-far unknown subcellular structures where these mRNAs and their protein products might play specific local functions. The cited study was only taking into account ~25% of the *Drosophila *genome, leaving considerable room to discover additional localizing transcripts and novel spatially restricted subcellular locations, which could unveil the existence of unknown subcellular compartments. Importantly, there was also a high correlation between the RNA localization sites and the localization of the proteins they encode, confirming that translation control is tightly regulated during RNA transport [19].

## 3. Localization and Translation of RNAs in Non-Polarized Cells

Recently, mRNA transport and localized translation have been found to occur in very specific regions within nonpolarized cells as well. In yeast, 423 mRNAs were found to localized to mitochondrion-bound polysomes [20]. In this case, about half of them encode putative nuclear-encoded mitochondrial proteins, suggesting that this serves to locally translate them in the vicinity of mitochondria [20]. Interestingly, the 3′-UTR of some of these mRNAs is sufficient to target the mRNAs to the vicinity of the mitochondria in a translation-independent manner [20, 21]. In another study, some mRNAs were found localized to the endoplasmic reticulum (ER), and further studies demonstrated that this localization can happen in a translation- and Signal Recognition Particle- (SRP-) independent manner [22]. In yeast, many mRNAs encoding peroxin or matrix proteins also colocalize with peroxisomes. For example, *PEX14* mRNA seems to localize to the peroxisomes and its 3′-UTR plays a role in this localization [23]. In conclusion, although localization of mRNAs around the mitochondria, ER, and peroxisomes was first thought to take place cotranslationally by the presence of specific import signals in the nascent proteins, it is becoming now clear that mRNAs have intrinsic targeting information for localization to the vicinity of these compartments.

Centrosomes and spindles also contain RNAs that either have structural functions or are carried along for asymmetric distribution during cell division. Ribosomes are also associated with spindles in frog egg extracts. Recently, Sharp and colleagues used high throughput sequencing to identify ~450 mRNAs significantly enriched on microtubules (MT-RNAs) [24]. They found an overrepresentation of transcripts involved in regulation of mitosis or playing roles in cell division, spindle formation, and chromosome function. This supports the notion that association of mRNAs with microtubules is a mechanism used to compartmentalize functionally related mRNAs also within the nucleocytoplasmic space of mitotic cells, where MT-RNAs are likely to contribute to spindle-localized mitotic translation.

## 4. Localization Coupled to Translational Control in Unicellular Organisms: The “Locasome” and the Ash1 Paradigm

**Figure 1:**
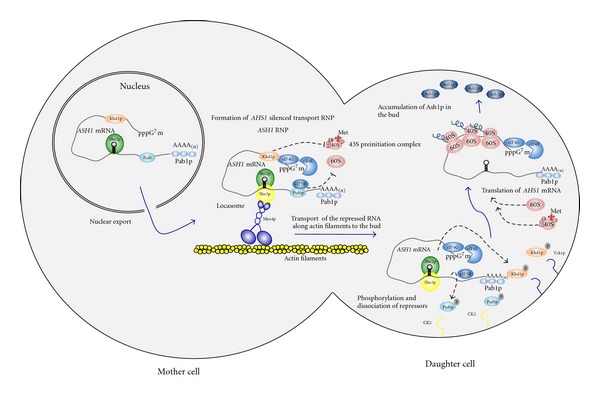
Transport and translation repression of *ASH1 *mRNA in* S. cerevisiae. ASH1 *mRNA is synthesized in the nucleus of the mother cell. The She2p protein is loaded onto* ASH1 *mRNA in the nucleus. Once in the cytoplasm, the *ASH1*-She2p complex binds to She3p which associates with Myo4p to form the transport machinery called the “locasome”. The translation repressors Puf6p and Khd1p and Pabp1 (which is needed for localization) are thought to be also loaded onto *ASH1 *mRNA before nuclear export. The locasome then transports silenced *ASH1 *RNPs to the bud through the actin filaments. Puf6p and Khd1p block *AHS1 *mRNA translation during transport by different mechanisms. One of them is through the interaction of Puf6p with eIF5B and further inhibition of the recruitment of the 60S ribosomal to the mRNA. Khd1p binds eIF4G. This interaction might prevent the recruitment of the 43S pre-initiation complex (consisting of the 40S subunit, the stabilizing factors eIF3, eIF1 and eIF1A and a ternary complex composed of eIF2 bound to an initiator Met-tRNA and GTP) to the mRNA, thereby blocking translation initiation. However, the exact mechanism is not clearly understood. Once the complex is localized to the bud tip, membrane associated kinases, CK2 and Yck1p, phosphorylate Puf6p and Khd1p respectively. This produces the dissociation of the repressors from *ASH1 *mRNA allowing thus translation activation. Ash1p then inhibits mating-type switching only in the daughter cell.

One of the best-characterized examples of RNA transport coupled to translation control is the localization of *Saccharomyces cerevisiae ASH1 *mRNA [25]. This mRNA is transported to the distal tip of the bud, resulting in the asymmetric sorting of the transcriptional repressor Ash1p into the daughter cell nucleus. In the daughter cell Ash1p represses transcription of the HO endonuclease, inhibiting mating-type switching in the daughter cell [26, 27]. Transacting factors Myo4p, She3p, and She2p drive *ASH1 *mRNA localization and form a complex known as the “locasome,” which is also essential for the localization of many other bud-localized mRNAs [28–30]. She2p is an RNA-binding protein that directly interacts with the *ASH1* mRNA *cis*-acting elements, and Myo4p is a type V myosin motor that functions to directly transport *ASH1 *mRNA to the bud along the actin cytoskeleton. She3p was initially suggested to act as an adaptor protein because it can simultaneously associate with Myo4p and She2p. However, recent data also suggested that She3p interacts directly with *ASH1 *mRNA [31], suggesting that it stabilizes RNP assembly through different interactions (Figure 1).

Silencing *ASH1 *mRNA before it is localized at the bud cortex in late anaphase is critical for asymmetric segregation of Ash1p to the daughter cell nucleus. Puf proteins are known to be versatile posttranscriptional repressors that can bind different transcripts with diverse cellular functions [32]. In yeast, Puf6p binds *ASH1 *mRNA and it is involved in translational repression of this mRNA and in its localization [33]. Deng et al. reported that Puf6p interferes with the conversion of the 48S preinitiation complex to the 80S initiation complex during translation initiation, and this repression is mediated through an interaction of Puf6p with the general translation factor eIF5B [34]. When the mRNA reaches the bud tip, protein kinase CK2 (casein kinase II) phosphorylates the N-terminal region of Puf6p and the repression is then relieved [34]. Khd1p is another protein that interacts with *ASH1 *mRNA and reduces translation initiation of the *ASH1 *mRNA [35, 36]. Several translation factors have been found to associate with Khd1p, including eIF4G1, eIF4G2, eIF4E, and PABP. Interestingly, Khd1p has been found to interact directly with the C-terminal domain of eIF4G1 to regulate the translation of *ASH1 *transcripts. Again, a phosphorylation step seems to trigger translational derepression at its final destination. At the bud plasma membrane, the type I Casein kinase (Yck1p) phosphorylates Khd1p. This leads to the dissociation of Khd1p from the *ASH1 *mRNA, releasing its translational repression [35] (Figure 1).

### 4.1. The Puf Family of Proteins, but Not the Locasome, Is Conserved

The adaptor proteins She2p and She3p link mRNAs to the myosin motor. They are only present in fungi, indicating that the main core of the “locasome” either evolved only in this lineage or was lost and further replaced by other machineries in other eukaryotes. Interestingly, members of the Puf family of proteins are present across kingdoms. *Drosophila melanogaster *has two Puf orthologs, vertebrates have three, yeast six*, Caenorhabditis elegans* 12, rice 19, and *Arabidopsis* 26 [32, 37]. Besides the aforementioned translation repression of *ASH1 *mRNA by Puf6, several other mechanisms of translation repression involving Puf members have been described. For example, yeast Puf5, Puf4, and Puf3, *D. melanogaster* Pumilio (Pum), *C. elegans *FBF and human Pum1 interact with the Ccr4-Pop-Not deadenylase complex, indicating that they influence translation and stability of their target mRNAs by controlling poly(A) tail length [38–42]. *Drosophila *Pum recruits the translation inhibitor 4E-HP to *hunchback *mRNA via the protein Brain tumor (Brat), thereby inhibiting translation initiation [43]. In *Xenopus, *Pum2 competes with eIF4E for cap structure binding and this also inhibits translation initiation [44]. Yeast Pufs function in mRNA localization; Puf5 is involved in the localization of *PEX14 *mRNA to the peroxisomes, and Puf3 drives mRNA localization to mitochondria [23, 45, 46]. Different classes of mRNAs have been found to be associated with different yeast Puf proteins. Puf3 binds mainly to nuclear mRNAs that encode mitochondrial proteins, Puf1 and Puf2 bind preferentially mRNAs encoding nucleolar ribosomal RNA-processing factors, and Puf5 associates with mRNAs that encode components of the spindle pole and chromatin modifiers [47]. This specificity of the interaction of a Puf family with subsets of functionally related mRNAs seems to indicate that different Puf families may regulate translation and localization of specific subsets of mRNAs. Mammalian Pum2 (mPum2) forms discrete RNA-containing particles in the somatodendritic compartment of polarized neurons, suggesting a role in localization of RNPs in dendrites and in the formation of stress granules [48]. In hippocampal neurons mPum2 is involved in translation repression of the mRNA encoding the translation initiation factor eIF4E and, interestingly, postsynaptic Pum also negatively regulates the expression eIF4E at the *Drosophila *neuromuscular junction (NMJ) [49, 50]. This suggests a conserved role of Pum proteins in regulating local translation at the synapses by controlling the local levels of eIF4E and thus general translation initiation on localizing mRNAs. Altogether, these observations support the notion that Pufs are conserved proteins that regulate localization and local translation of different mRNAs.

## 5. Localization-Coupled Translational Control in Multicellular Organisms: The Bic-D/Egl mRNA Localization Machinery and the *Osk* Paradigm

**Is There a General Drosophila RNA Localization Machinery?**

In *Drosophila, *an RNA transport machinery plays a key role in oogenesis by localizing into the oocyte RNAs required for oocyte determination, differentiation, and formation of anterior-posterior and dorsal-ventral polarity. This machinery is composed of Bicaudal-D (Bic-D) and Egalitarian (Egl) proteins, which interact with the cytoplasmic microtubule motors Dynein(Dyn)/Dynactin to move the mRNA cargo on microtubules (MTs) to distinct cellular compartments [51, 52]. During oocyte determination, a single cell among an interconnected cyst of sixteen germline cells differentiates into an oocyte, and this process involves the preferential accumulation of specific messenger RNAs and proteins in this cell. The other fifteen cells adopt a nurse cell fate and provide the oocyte with the materials required for oocyte growth. *Bic-D *loss-of-function mutant females produce egg chambers composed of 16 polyploid cells with nurse cell appearance, indicating that the oocyte fails to differentiate. Since *Bic-D *mutant egg chambers fail to accumulate oocyte-specific mRNAs (such as *osk*, *orb, Bic-D, *and *fs(1)K10*) in the future oocyte, it is suggested that the loss of oocyte differentiation is due to a failure in the transport of oocyte-specific proteins and mRNAs from the nurse cells into the oocyte [53, 54]. Ovaries mutant for *egl *as well as wild-type ovaries treated with microtubule-depolymerising drugs show the same 16 nurse cell phenotype as *Bic-D *mutants [55, 56]. Studies using fluorescently labelled mRNAs injected into the nurse cells have shown that Bic-D and Egl are recruited to injected *grk *and *bcd *mRNAs in the nurse cells, and these proteins are required for *grk *transport into the oocyte [57]. The same studies found that transport along MTs via Dyn is also required for the efficient transport of *grk*, *bcd*, and *osk *RNA from the nurse cells to the oocyte [57]. Moreover, the Bic-D/Egl/Dyn machinery is also used for the apical localization of *inscuteable *mRNA in neuroblasts [58] and pair rule and *wingless *segmentation mRNAs in the blastoderm embryos [59].

The formation of the Bic-D/Egl/Dyn complex has been studied in *Drosophila *and in mammals. While *Drosophila *Egl interacts directly with Bic-D and also binds the Dyn light chain (Dlc), mammalian orthologues of Bic-D bind *in vitro *directly to components of the Dyn and Dynactin complexes and they also associate *in vivo *with them [60, 61]. Therefore, it is suggested that the Bic-D/Egl complex acts as a link between a microtubule-dependent Dyn motor and the mRNAs. Dienstbier et al. showed that Egl binds directly to mRNAs that localize in the oocyte and apically in the embryos, suggesting that Egl is the factor that links the molecular motors and Bic-D with the transported mRNAs [62]. However, it is still not clear whether Egl is a general link for all mRNAs transported by this machinery or whether additional proteins are required for the specificity of the interaction since, so far, only a specific direct link between the complex Bic-D/Egl and the localization signals of *grk, K10,* and *I factor *mRNAs have been demonstrated. Moreover, Egl alone seems to have an inherent degree of mRNA promiscuity *in vivo *and *in vitro *[62, 63].

The current model proposes that all maternally localized mRNAs are transported by the Bic-D/Egl/Dyn localization machinery from nurse cells to the oocyte [10]. The current data also suggest that Bic-D and Egl form part of a general mRNA transport machinery used repeatedly throughout *Drosophila *development. Although many mRNAs are transported by this machinery, studies of the proteins controlling their translation while transported are still missing for most of the localized transcripts. Nevertheless, in a similar way to the yeast locasome, the Bic-D/Egl/Dyn machinery must also be part of a bigger RNP complex that contains proteins involved in translation control of the transported mRNAs, ensuring that protein synthesis is only activated once the mRNAs reach their final destination.

**Control of Translation of RNAs Transported by the Bic-D/Egl Machinery: The *Osk* Paradigm**

**Figure 2:**
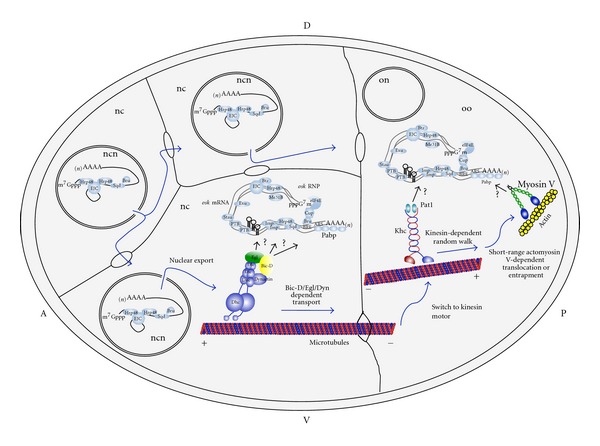
Transport and translation repression of* osk *mRNA during* Drosophila *oogenesis*. osk *mRNA is synthesized in the nucleus (ncn) of the nurse cells (nc) and exported already as a complex with several factors controlling its transport and/or translation (light blue circles), like the exon junction complex (EJC, composed of Mago-Nashi/Y14/eIF4AIII), Hrp48, Bru and Sqd. In the nc cytoplasm more factors controlling translation (Me31B, Cup, Bru, PTB, Imp), localization (Stau, Exu, Sqd, Btz, Pabp) or stability (Pabp) (light blue circles) associate with *osk *mRNA to form a big RNP complex (light blue circles). This RNP contains many *osk *mRNA molecules and multiple factors that repress translation of *osk *by several different mechanisms (see text for details). This big silenced *osk *RNP is recruited by the Bic-D/Egl/Dyn localization machinery which directs its minus end directed microtubule transport in the nurse cell cytoplasm and through the ring canals into the oocyte (oo). Factors linking *osk *RNPs to the transport machinery are not known. Since Egl binds directly some other localized mRNAs, Egl may be the linking factor. Other proteins in complex with Bic-D, such as Pabp, (which binds directly to *osk *mRNA through adenine rich sequences (ARS) and the poly-(A) tail) could also be involved. Within the oocyte the silenced *osk *RNP is then transported by a kinesin motor probably by a random walk process in a poorly polarized microtubule network with a net movement toward the posterior cortex. This movement is followed by a short-range actomyosin-dependent transport or entrapment of *osk *mRNA to the posterior cortex. During its journey *osk *mRNA associates with the factors repressing its translation. Although different proteins may associate with *osk *during different stages of oogenesis, most of them are probably associated with it during its all trip to the posterior. When *osk *mRNA reaches the posterior cortex at stage 9 of oogenesis, translation repression is relieve and the mRNA gets translated (not shown in this figure).

*Drosophila osk *mRNA is transported by the Bic-D/Egl/Dyn transport machinery from nurse cells to the oocyte [57, 59] (Figure 2). Within the oocyte *osk *mRNA switches to a kinesin-based motor that transports it to the posterior cortex. However, only kinesin heavy chain (KHC), but not the kinesin light chain (KLC), is required for this movement, and the KLC-likeprotein PAT1 functions as a positive regulator of KHC during posterior localization of* osk *mRNA [64–66]. Although the mechanism of localization to the posterior has been controversial, based on recent studies that followed the movement of *osk *mRNA particles *in vivo *Zimyanin et al. proposed a new model where *osk *mRNA is localized by random walk on microtubules. Each particle undergoes large numbers of active movements in different directions, but shows an excess of movements towards the posterior which is sufficient to produce the strong posterior localization seen by stage 9 [67]. While kinesin is involved in this long-range MT-based transport of *osk *mRNA throughout the oocyte and into the posterior cytoplasm, recent results indicate that this movement is followed by short-range actomyosinV-dependent translocation or entrapment of *osk *mRNA at the posterior cortex [68].

*osk *mRNA is one of the most studied models for translation control during transport, and the aforementioned transport machineries must associate with different factors that control translation of the mRNA during transport (Figure 2). During its extended journey, *osk *mRNA translation must be repressed since Osk protein is only observed once the mRNA reaches its final destination at the posterior cortex of the oocyte after stage 8 of oogenesis. Mutants in *armitage *(*armi*), *aubergine *(*aub*, also known as *sting*), *spindle-E *(*spn-E*, also known as *homeless *(*hls*)), *maelstrom *(*mael*) [69], *zucchini *(*zuc*), *squah *(*squ*) [70], and *krimper *(*krimp*) [71] show premature translation of *osk *mRNA in the oocyte during the first part of oogenesis up to stage 6. Interestingly, *spn-E*, *armi*, *aub*, *zuc, *and *squ *are also needed for silencing of the *Stellate *locus, a gene regulated endogenously by small RNAs [70, 72–75]. *spn-E*, *zuc, aub*, *squ *and *krimp *are additionally needed for silencing of retrotransposons in the *Drosophila *germline [70, 71, 76, 77]. Silencing of the transposable elements and the *Stellate *locus is achieved by a mechanism that uses a class of small RNAs called repeat-associated small interfering RNA (rasiRNAs), subsequently renamed Piwi-interacting RNAs (piRNAs) [78–80]. The *mael, krimp, spn-E *[71], *zuc, squ, aub, *and *spn-E *[70] genes have been implicated in the production of these piRNAs. This pathway is germline-specific and depends on the Piwi subfamily of argonaute proteins, which include Aub, Piwi, and Ago3 (reviews in [79, 80]). It is therefore possible that translational silencing of *osk *mRNA during early oogenesis is driven by piRNA-Piwi-Argonaute complexes interacting with *osk *mRNA. However, whether piRNAs play a direct or indirect role in translation control and which piRNAs are involved in translation repression of *osk *mRNA should still be studied. Egg chambers mutants in the *Maternal expression at 31B *(*Me31B*) gene show ectopic Osk accumulation in the nurse cells rather than in the oocyte during early oogenesis indicating that Me31B repress *osk *translation during its transport through the nurse cell into the oocyte [81]. Since Me31B egg chambers degenerate around stage 6, a role in translation repression in later stages could not be ruled out. *Drosophila* PTB (polypyrimidine tract-binding protein) is also involved in translational repression during early (starting at stage 5-6) and late oogenesis of the localizing *osk *mRNA by binding along the *osk *3′-UTR and mediating assembly of high-order complexes containing multiple *osk* RNAs that produce translational silencing [82]. Interestingly, a 50 kDa pumpkin phloem RNA-binding protein (RBP50), which is evolutionarily related to animal PTBs, seems to be part of the core of an RNP that contains proteins and RNAs transported in the phloem [83]. A complex made up by Bruno (Bru) and Cup represses *osk *mRNA cap-dependent translation from stages 5-6 on [84]. Bru binds simultaneously to Bruno-response elements (BREs) in *osk *3′-UTR and to Cup. Cup is an eIF4E-binding protein that competes with eIF4G for the binding to eIF4E, thereby inhibiting recruitment of the small ribosomal subunit to *osk *mRNA [84]. Egg chambers expressing a Cup mutant protein that cannot bind eIF4E show precocious expression of *osk *mRNA in stages 6 to 9 and also increased expression in stage 9 oocytes. Another mechanism, independent of the Cup-eIF4E interaction but dependent on Bru, also drives translation repression during mid oogenesis. This mechanism involves the formation of *osk *mRNA oligomers by binding of Bru that produces the formation of large (50S-80S) silencing particles that cannot be accessed by ribosomes [85]. Hrp48 binds sequences in the *osk *5′ and 3′UTRs and has also been involved in localization and translational repression of *osk *mRNA after stage 9 of oogenesis, although how Hrp48 regulates translation is still not known [86].

Interestingly, Cup was shown to be also involved in translational repression of *grk *mRNA, which is also transported by the Bic-D/Egl complex. Based on genetic and biochemical interactions studies, Clouse et al. proposed a model for translation regulation of* grk *mRNA [87]. In their model, Cup and Bruno also function in complex with Sqd, Otu and Hrb27C/Hrp48 in repressing translation of *grk *mRNA before it is localized. While this is not proven yet, this repression would also appear to act at the level of translation initiation. They also showed that before the RNA has reached its final destination in the future dorsal-anterior region of the oocyte, a well-established translation factor, the poly(A)-binding protein (PABP), functions with Encore (Enc) to facilitate the translational activation of *grk *mRNA [87].

Our group recently reported that *Drosophila pabp *genetically interacts with *Bic-D *and that the two proteins form an RNA-dependent complex. *pabp* mutants show reduced *osk* mRNA stability and display defects in *osk* mRNA localization during early oogenesis. These findings demonstrated that PABP plays a key role in *osk* mRNA localization and is also essential in the germline for oocyte growth [88]. The recent finding that mammalian PABP can bind to microtubules [89] also hints that PABP links *osk* mRNA to the transport machinery in addition to controlling its RNA stability during transport. Although it seems that PABP is not involved in controlling translation during early oogenesis, a study of the role of PABP in activating translation of *osk* mRNA after it has reached its final destination is still missing due to the lack of *pabp* mutants that specifically affect late oogenesis. Another factor that may play a role in controlling translation and localization of both *grk* and *osk* mRNAs is the insulin-like growth factor II mRNA-binding protein (IMP) [90, 91]. However, genetic studies so far failed to reveal such a requirement for IMP, indicating that its function is at best a redundant one [90, 91]. In summary, Cup, PABP, IMP, Bruno, and Hrp48 are factors that can associate with the Bic-D/Egl/Dyn transport machinery to regulate the fate and translation of specific transported mRNAs.

**How Conserved Is the Bic-D/Egl Complex across Eukaryotes?**

Studies on the functional role of Bic-D homologs in different species suggest that Bic-D proteins are coiled-coil proteins that function as factors linking the Dyn/Dynactin minus-end-directed motor complex with different cargos [52]. Besides its role in the aforementioned Bic-D/Egl/Dyn RNA transport machinery, *Drosophila *Bic-D is also involved in lipid droplet transport [92], migration of photoreceptor cell nuclei [93], movement of the oocyte nucleus [94], transport of Chc and synaptic vesicle recycling at the neuromuscular junction [95]. *Drosophila *Bic-D also binds an RNA binding protein, the mental retardation protein (FMRP), and both are required for efficient branching of the dendritic arbour [96]. In mammals, Bic-D proteins are required for anchoring the centrosomes to the microtubules [97]. Mammalian Bic-D2 associates with RanBP2, a component of the nuclear pore complex, and is needed to regulate centrosome and nuclear positioning during mitotic entry [98]. By binding Rab6, mammalian Bic-D also controls COPI-independent Golgi-ER transport [99], and Rab6B-Bic-D1 interaction regulates retrograde membrane transport in neurites of human neuronal cells [100]. Like *D. melanogaster *Bic-D, *C. elegans *Bic-D is also involved in nuclear migration [101] and in dendritic branching [102]. Altogether, these studies show that Bic-D acts as a modulator of the Dyn transport complex in different organisms, linking different, but sometimes conserved cargos, such as mRNAs, nuclei, and vesicles.

The *Bic-D *gene is conserved throughout the animal kingdom, but is not present in plants and fungi. While there is only one gene encoding Bic-D in insects, *C. elegans *and the ascidians *Ciona intestinalis *and *Ciona savignyi*, the gene is duplicated in higher vertebrate lineages, including mammals (human, mouse, and gorilla) and birds (chicken). Accordingly, the two homologs of *Drosophila Bic-*D were named *Bic-D1 *and *Bic-D2*. In the amphibian *Xenopus, *one *Bic-D1 *and two *Bic-D2 *homologs are found. Interestingly, fishes (*Danio rerio, Gasterosteus aculeatus, Oryzias latipes, takifugu rubripes, Tetraodon nigroviridis*) have two homologues of the *Bic-D1 *gene and two homologs of *Bic-D2*. In addition, in fishes there is also a third, deeply divergent gene, probably representing an ancestral version of the *Bic-D* gene. In the sea lamprey *Petromyzon marinus *there are also two *Bic-D *genes, one *Bic-D1* ortholog and one that also seems to be close to the original ancestor *Bic-D *gene (taken from http://cegg.unige.ch/orthodb [103]).

Recent studies in the wasp *Nasonia vitripennis *point to a conserved role for Bic-D in mRNA localization in nondipteran insects [104]. Knocking down *Bic-D* by RNAi in the *Nasonia *germ line produced oogenesis phenotypes similar to the ones observed in *Drosophila Bic-D *mutants. More importantly, mRNAs that localize to the *Nasonia *oocyte also fail to localize to their normal destination in *Bic-D *loss-of-function animals. These studies strongly suggest that the role of Bic-D in the localization of oocyte determinants, which also involved the organization of a polarized microtubule network, is conserved between *Nasonia *and *Drosophila. *Even though these insects share a similar germ line development, evolutionary they diverged over 200 million years ago. Thus, although no other examples of Bic-D-dependent mRNA transport in other species have been investigated, the high conservation of Bic-D proteins in the animal kingdom suggests that Bic-D proteins have played a conserved role in mRNA transport during evolution. The study of the biological roles of Bic-D in different eukaryotes is an interesting field that deserves further investigation.

In contrast to *Bic-D, egl *is not present in mammals, and only one homolog is found in *D. melanogaster *and *C. elegans. *So far, functional studies on *egl *have been restricted to *D. melanogaster, *but recent studies on the giant shrimp (*Penaeus monodon) egl *ortholog suggested an involvement in ovary development as well [105]. Since *Drosophila *Egl is an adaptor that binds directly to localization signals in mRNAs, most likely Bic-D/Egl complexes function in mRNA localization only in Arthropoda and Nematoda, and it appears that other adaptor proteins not related or only distantly related to Egl may link the Bic-D/Dyn localization machinery to localizing mRNAs in other phyla.

Bic-D is highly conserved across the animal kingdom and other highly conserved RNA-binding proteins that play roles in RNA localization, such as PABP [88], FMRP [96] and other proteins (Vazquez and Suter, unpublished results) are also present in Bic-D complexes. This suggests that different adaptor proteins may be linking the transported mRNAs to the transport machinery in a species-specific manner, as well as in a mRNA- or tissue-specific manner. One of these proteins might be PABP. Cytoplasmic PABPs are general translation factors and are conserved throughout eukaryotes [106]. One cytoplasmic *pabp *gene is present in the unicellular fungi *Candida albicans, Saccharomyces cerevisiae, *and *Schizosaccharomyces pombe*, as well as in *D. melanogaster*. In contrast, vertebrates contain multiple cytoplasmic PABPs. They include PABP1 (also known as PABP, PAB1, PAB, and PABPC1), PABP4 (also called PABPC4, iPABP or APP-1), ePABP (embryonic PABP), ePABP2, and the mammalian-specific tPABP (testis-specific PABP, also called PABPC2 in mouse and PABPC3 in humans) [107, 108]. A shorter version of these PABPs called PABP5 or PABP5C is also present in higher eukaryotes, and it is highly conserved in primates, rodents, and humans [109]. Counting all the family members, eight genes are present in *Arabidopsis*, four in *Zebrafish*, Chicken, and *Xenopus Tropicalis, *seven in humans, and six in mouse (taken from http://cegg.unige.ch/orthodb [103, 110]). To date, most of the functional studies have been focused on the prototype PABP1; however, the versatility and the high number of genes encoding PABP family members open the possibility that different PABPs may regulate localization and/or translation of different transported mRNAs. A conserved role for PABP in RNA localization is supported by the recent finding that PABP binds directly or in a complex with other proteins to non-poly(A) sequences in the *osk*, *bcd, *and *Vasopressin* mRNAs, which are essential for correct localization of these transcripts in *Drosophila *oocytes and mammalian dendrites, respectively [88, 111–114]. Furthermore, yeast Pab1p is required to restrict *ASH1 *mRNA to the bud tip, indicating that the role of these proteins in RNA localization is also conserved in unicellular eukaryotes [115].

FMRP is also a highly conserved protein, displaying 92% amino acid identity between humans and chicken [116]. In humans and mouse there are three paralogous proteins (namely, FMRP, FXR1 and FXR2) [117–120]. The three genes have a conserved gene structure suggesting they may be derived from a common ancestor [120]. Zebrafish possess also three FMR1-related genes that are orthologous to the human and murine ones [121]. In *Drosophila* there is only one single orthologous gene that has higher overall similarity to human FXR2 than to FMR1 or FXR1 [122]. Several lines of evidence prove that FMRP orthologs are involved in RNA localization and translation control [123]. FMRP colocalizes and immunoprecipitates with several dendritically localized mRNAs in mammalian neurons [123]. FMRP knock down mice show an excess of protein synthesis and loss of stimulus-induced translation of some localized mRNAs as well as a failure to augment trafficking of certain mRNAs in neurons upon mGluR activation, indicating that FMRP is crucial for transport and regulation of local translation of certain mRNAs at the synapses [123]. The recent finding that *Drosophila *FMRP binds to Bic-D and that both cooperate to control dendrite morphogenesis [96] and that FMRP controls RNA transport in neurons [124] suggests that the role of FMRP as a link between the transport machinery and localizing neuronal mRNAs may be conserved between mammals and *Drosophila*.

Another protein that is required for translational control of localized *osk *and* grk *mRNAs is the insulin-like growth factor II mRNA-binding protein (IMP) [90, 91]. Preliminary results from our lab indicate that *Drosophila *IMP is a component of the Bic-D/Egl complex (Vazquez-Pianzola, Bullock and Suter, unpublished). IMP is highly conserved in the animal kingdom. Most likely the vertebrate IMP family originated by repeated gene duplications shortly after the divergence of vertebrates from other major metazoan clades. This is supported by the finding that *D. melanogaster, C. elegans* and the ascidians *C. intestinalis, and C. savignyi *have only one gene, whereas most vertebrates possess more than one ortholog. In most mammals (i.e., human, rat, mice), birds (Chicken), and reptiles (*Anolis Carolinensis*), three IMPs (namely, IMP1, IMP2 and IMP3) are present. Interestingly, *Gorilla* and the fish *D. Rerio *have four orthologous genes, the additional one being most closely related to mammalian IMP2. Mammalian IMP1 is most closely related to *Drosophila* IMP. The amphibian *Xenopus Tropicalis* contains only one IMP gene, homologous to mammalian IMP3, which was originally named Vg1 RNA-binding protein (Vg1RBP/Vera) (Taken from http://cegg.unige.ch/orthodb [103, 125]). These proteins are paradigms of RNA binding proteins required for transport and local translation of RNAs. The chicken IMP1, also known as the zipcode-binding protein (ZBP-1), is required for beta-actin mRNA localization and translational repression during transport to the leading edge of motile fibroblasts and neurons, while the Vg1RBP/Vera is required for Vg1 mRNAs localization to the vegetal Pole of the *xenopus* oocytes during maturation [28, 125]. These observations show that IMP proteins play a function that has been conserved during animal evolution.

## 6. Concluding Remarks

Many studies have been performed on the factors regulating translation of specific mRNAs while transported to their destination. One conclusion from these reports is that many of them, such as Pufs, FMRPs, IMP and PABP proteins, have been highly conserved during evolution and that their roles in translation also seem to be conserved across eukaryotes. The “locasome” in yeast and the Bic-D/Egl localization machinery in *Drosophila *seem to be general links between the RNA-transporting molecular motors and the translation machinery, acting either via myosin or Dyn/Dynactin motors. The “locasome” seems to be only present in unicellular fungi while Bic-D proteins are conserved in the animal kingdom. Thus, further studies of Bic-D proteins in RNA transport in other animals, including humans, will shed light on the question if the mechanisms of RNA transport are indeed conserved over the entire animal kingdom.

The reports that even in non-polarized cells mRNAs are localized to different compartments, such as the vicinity of mitochondria, peroxisomes, spindles or ER, raise the question of how these mRNAs are transported. Regarding this, it is known that some mRNAs are localized independent of translation and that 3′-UTR regions of many mRNAs are involved in their localization to these subcellular compartments. However, only few studies of the factors involved in these processes have been performed. Another intriguing question that remains to be investigated is whether mRNA localization to the vicinity of these organelles involves an active transport or just a diffusion mechanism followed by anchoring of the mRNAs through factors, such as specific RNA binding proteins localized to these structures. Thus, high-throughput *in situ *hybridization screens and proteomics approaches of different subcellular fractions are needed to shed light on the existence of new subcellular compartments and the common features of the RNAs targeted to them. In plants, some RNAs travel between cells and in the phloem, but the study of the factors controlling their transport and translation is still scarce. Neither the locasome nor the Bic-D transport machineries are conserved in plants, pointing to novel, so-far unknown RNA transport players awaiting discovery in these organisms. Extensive studies on mRNA translation should also be done in non-model organisms. To our knowledge, nothing is known about subcellular localization of RNAs and translational control in protists, even though examples of localized mRNAs have been described in bacteria, animals, fungi and plants. This strongly suggests that subcellular localization of mRNAs is an essential process that most likely is required for most forms of life, and that the mechanisms of subcellular localization of RNAs were conserved during evolution. It is worth testing whether this process also functions in archeal lineages, since some of them are believed to be current representatives of the eukaryotic ancestors.

Many of the proteins controlling translation during transport of their target mRNAs seem to repress translation at the level of translation initiation either competing for the formation of the eIF4E complex or inhibiting 60S subunit joining. This makes sense, since translation initiation is the limiting step in all the translation process indicating that it must be tightly regulated. Phosphorylation of the yeast translational repressors Puf6 and Khd1p and the chicken ZBP by specific kinases localized at the mRNA final destination is involved in local translational de-repression of their targets [34, 35, 126]. The presence of similar mechanisms of translation control of localized mRNAs in unicellular and higher eukaryotes, may indicate that the control of translation initiation, the presence of locally expressed kinases and the phosphorylation status of the RNA-binding proteins are conserved features used for the RNA localization machineries during evolution to control translation of localized mRNAs.

Elucidating the global composition of different RNP-complexes and identifying the factors that are common and the ones that are specific to sort individual mRNAs to the different subcellular compartments is an interesting and important question for future research in the field. Similarly, elucidating in detail the mechanisms that are in place to couple mRNA localization to local protein synthesis across different eukaryotes is another fascinating question to tackle.
